# [Retracted] BAMBI inhibits inflammation through the activation of autophagy in experimental spinal cord injury

**DOI:** 10.3892/ijmm.2026.5872

**Published:** 2026-05-25

**Authors:** Yin Yang, Chunyang Guo, Bo Liao, Junjun Cao, Chen Liang, Xijing He

Int J Mol Med 39: 423-429, 2017; DOI: 10.3892/ijmm.2016.2838

Following the publication of the above article, a concerned reader drew the Editor's attention to the fact that the Nissl staining images shown in Fig. 3C on p. 427 contained a series of internally duplicated groupings/repeated patternings of cells within the various data panels that could not easily be attributed to coincidence. A subsequent investigation of the data in this paper revealed that the Nissl staining images shown in Fig. 2C were similarly affected by the same phenomenon.

After having conducted an internal investigation of the data in this paper, the Editor of *International Journal of Molecular Medicine* has decided that this article should be retracted from the publication on the grounds of an overall lack of confidence in the presented data. The authors were asked for an explanation to account for these concerns, but the Editorial Office did not receive a reply. The Editor sincerely apologizes to the readership for any incovenience caused, and we thank the reader for drawing this matter to our attention.

